# 2738. Two-step sulfonamide antibiotic challenges in patients who require treatment with trimethoprim-sulfamethoxazole at an academic medical center: Outcomes in a largely immunocompromised population

**DOI:** 10.1093/ofid/ofad500.2349

**Published:** 2023-11-27

**Authors:** Aya Samhan, Shyam Joshi, YoungYoon Ham

**Affiliations:** OSU/OHSU, Portland, Oregon; Oregon Health & Science University, Portland, Oregon; Oregon Health & Science University, Portland, Oregon

## Abstract

**Background:**

Sulfonamide antibiotic allergy is the second most reported medication allergy. The allergy label limits the use of trimethoprim-sulfamethoxazole (TMP-SMX), the antibiotic of choice for pneumocystis jirovecii pneumonia (PJP), Toxoplasma, and many types of urinary tract infections. Immunocompromised patients, including solid organ transplant recipients, are considered to be at a higher risk for developing an infection. While penicillin allergies are commonly tested and patients delabeled, sulfonamide challenges are not as prevalent. Patients who are tested following a transplant may be on medications that lower the negative predictive value of the challenge, raising questions about whether they truly passed the challenge or whether mild reactions were masked.

**Methods:** Thirty-seven patients labeled with a sulfonamide allergy who were evaluated and challenged with two doses of TMP-SMX were included. Adults (≥18 years) with a sulfonamide antibiotic allergy were evaluated and challenged between June 2020 and May 2023. Data collection included the number of patients challenged and delabeled, number of patients who had reactions and what type of reaction, and proportion of patients who completed the treatment or prophylaxis course for which TMP-SMX was the first-line antibiotic.

**Results:**

Thirty-seven patients were challenged, of which twenty-seven patients were immunocompromised (73%). Thirty-four (92%) passed the sulfonamide challenge and were delabeled, and thirty-two (86%) completed the full course of TMP-SMX prophylaxis or treatment. Nineteen patients (51%) were tested following immunosuppression or antihistamine treatment due to a new transplant. Five patients were not able to be delabeled due to mild or delayed reactions to the challenge or treatment, including two patients that passed the sulfonamide challenge and were delabeled but had a mild reaction during SMX-TMP therapy and were relabeled.

**Conclusion:**

The benefits of sulfonamide antibiotic allergy testing can have great effects in immunocompromised patients in preventing the use of second-line antibiotics. Sulfonamide challenges were shown to be safe and effective in immunocompromised patients in delabeling the sulfonamide antibiotic allergy and leading to the completion of treatment with TMP-SMX.

**Disclosures:**

**Shyam Joshi, MD**, Cogent: Honoraria|Leo Pharma: Honoraria|Nectar Allergy: Advisor/Consultant|Nectar Allergy: Stocks/Bonds|Sanofi/Regeneron: Advisor/Consultant|Sanofi/Regeneron: Honoraria|Takeda: Honoraria

